# Associations Between Sociodemographic Characteristics, Pre Migratory and Migratory Factors and Psychological Distress Just After Migration and After Resettlement: The Indian Migration Study

**DOI:** 10.4103/0971-9962.162028

**Published:** 2015-08-03

**Authors:** Sutapa Agrawal, Fiona C Taylor, Kath Moser, Gitanjali Narayanan, Sanjay Kinra, Dorairaj Prabhakaran, Kolli Srinath Reddy, George Davey Smith, Shah Ebrahim

**Affiliations:** 1 Centre for Control of Chronic Conditions, Public Health Foundation of India, New Delhi, India; 2 Departments of Epidemiology and Population Health and Cochrane Heart Group, London School of Hygiene and Tropical Medicine, London; 3 Departments of Non-communicable Disease Epidemiology, London School of Hygiene and Tropical Medicine, London; 4 Centre for Chronic Disease Control, New Delhi, India; 5 Public Health Foundation of India, New Delhi, India; 6 School of Social and Community Medicine, University of Bristol, Bristol, United Kingdom

**Keywords:** India, Indian Migration Study, migrants, psychological distress, rural-urban migration

## Abstract

**Background/Objectives:**

Migration is suspected to increase the risk for psychological distress for those who enter a new cultural environment. We investigated the association between sociodemographic characteristics, premigratory and migratory factors and psychological distress in rural-to-urban migrants just after migration and after resettlement.

**Methods:**

Data from the cross-sectional sib-pair designed Indian Migration Study (IMS, 2005–2007) were used. The analysis focused on 2112 participants aged ≥18 years from the total IMS sample (*n* = 7067) who reported being migrant. Psychological distress was assessed based on the responses of the 7-questions in a five-point scale, where the respondents were asked to report about their feelings now and also asked to recall these feelings when they first migrated. The associations were analyzed using multiple logistic regression models.

**Results:**

High prevalence of psychological distress was found just after migration (7.3%; 95% confidence interval [CI]: 6.2–8.4) than after settlement (4.7%; 95% CI: 3.8–5.6). Push factors as a reason behind migration and not being able to adjust in the new environment were the main correlates of psychological distress among both the male and female migrants, just after migration.

**Conclusions:**

Rural-urban migration is a major phenomenon in India and given the impact of premigratory and migratory related stressors on mental health, early intervention could prevent the development of psychological distress among the migrants.

## Introduction

Migration is the process of social change whereby an individual moves from one cultural setting to another for the purposes of settling down either permanently or for a prolonged period.[1] The physical act of residential relocation is of brief temporal duration but the processes of absorption or assimilation, which follow in the wake of migration, may take many years before resultant tensions are resolved and the individual migrants learn to cope effectively with the new environment so as to become a functional member of the recipient community.[1] The process is inevitably stressful, and stress can lead to mental illness.[2] Studies in the west showed that migration may have negative health consequences such as increased risk of depressive and anxiety disorders due to physical and psychosocial strains experienced by migrants throughout the migration process.[3]

Migration that deals with the moving of people from one particular geographical area to another has long been under investigation in relation to its impact on mental health of the migrating people.[4–7] Increasing rates of migration throughout the world have led to a growth of interest in its impact on migrants’ mental health.[8] Several studies showed that rate of common mental disorders are higher among migrating groups and groups with out-migration (Kimura and Mikolashek, 1975;[9] Krupinski, 1967). It has been argued that consequences of migration and resettlement pose certain threats to the psychological well-being of the migrants due to accompanied changes in their physical and psychosocial environment.[10–12] The psychosocial factors that might be influenced by migration, and thereby pose a negative effect on mental health are social support, social participation and feeling of powerlessness.[13] Problems such as feeling loneliness, helplessness, frustration, increased household and social burdening are common among the migrants.[7]

India has experienced a large scale rural to urban migration over the last three decades which may have put excess stress on individuals and their families. In 2001, 309 million persons were migrants in India based on place of last residence, which constitute about 30% of the total population of the country. This figure indicates an increase of around 37% from Census 1991 which recorded 226 million migrants. Internal migration is now recognized as an important factor in influencing social and economic development, especially in developing countries.[14] Though in all censuses, rural to rural migration stream has been the most important in India, the Census of India acknowledges rural-urban migration as one of the important factors contributing to the growth of urban population. The migration data of 2001 Census indicate that 20.5 million people enumerated in urban areas are migrants from rural areas who moved in within the last 10 years. It may also be worth noting that rural-urban migration constitutes a significant component of inter-state migration (about 41.1 million as of 2001) taking place within the country.

Traditional rural to urban migration exists in India as villagers seek to improve opportunities and lifestyles. The scope and magnitude of rural to urban migration streams within India and many other regions of the world are well documented but little empirical evidence exists on the knowledge about the processes affecting the rural migrants into urban, industrial communities, and the impact of migration on the mental health of migrants. In this study, we investigated the association between sociodemographic characteristics, premigratory and migratory factors and psychological distress of migrants just after migration and after their resettlement.

## Methods

### Study design

Data from the Indian Migration Study (IMS) conducted during 2005–2007 were used for this study. The design and sampling methodology of the IMS has been described previously.[15–17] Briefly, the IMS is a cross-sectional sib-pair study, part of a larger cardiovascular risk factor surveillance system[18] in industrial populations all over India. The IMS was carried out in factory settings in four cities from northern, central and southern India (Lucknow, Hindustan Aeronautics Ltd.; Nagpur, Indorama Synthetics Ltd.; Hyderabad, Bharat Heavy Electricals Ltd.; and Bangalore, Hindustan Machine Tools Ltd). Information on rural-to-urban migration was solicited from factory workers and their co-resident spouses. Factory workers who had migrated from rural to urban areas, along with a 25% random sample of urban nonmigrants, were asked to participate in the study. Each migrant participant was asked to identify a nonmigrant sibling residing in a rural area, preferably of the same gender and close to them in age, who was then also invited to participate in the study. In a small number of cases where no rural sibling was available (<5%), a cousin or a close friend from the same village was invited. There were no other exclusion criteria at this recruitment stage. This convenience sampling strategy resulted in rural dwelling siblings being drawn from anywhere in the country (18 of the 28 states), reflecting the migration patterns of the factory workers and their spouses. A substantial proportion came from the four large states in which the factories were based. The urban participants were also asked to identify a nonmigrant, urban dwelling sibling for inclusion in the study.

### Measurements

#### Psychological distress

Psychological distress was assessed based on the responses of the 7-questions, in which all respondents were asked to report about their feelings now and also asked to recall these feelings when they first migrated. The questions specifically asked was: About your feelings now, how often do you feel and still thinking back to when you first moved to the town/city, did you feel: (a) Insecure, stressed or anxious (b) frightened (c) tearful (d) sleepless (e) loss of appetite (f) loss of interest in usual activities and (g) difficulty in concentrating. The responses were coded in a 5-point scale (1 = not at all, 2 = rarely, 3 = sometimes, 4 = often, and 5 = all the time). A score of 0 was given if reported not at all or rarely or sometimes to these questions and 1 was given for often or all the time for each of the above 7 items. The scores were then combined together and computed to form total psychological distress scores, which ranged from 0 to 7, which was further categorized as 0 (absence of psychological distress) and 1 or more (presence of psychological distress). High internal consistency (Cronbach’s Alpha Statistics) of this instrument is reported, with a slight difference for internal reliability of items for computation of the scores for just after migration (Cronbach’s alpha value = 0.7063) and after resettlement (Cronbach’s alpha value = 0.5258).

We studied premigratory and migratory factors as a covariate for mental distress, classified in terms of: Reasons for migration, percentage of life lived in an urban area, when the spouse joined migrant, acceptance in workplace, and adjustment in the urban environment. With migration being one of the important factors contributing to the growth of urban population, we explored whether it is push (out of the rural area) or pull (toward the urban area due to its perceived benefits) explains migration in India (Appendix 1 for push and pull factors of migration).

Participants were also asked to complete an interviewer-administered questionnaire to gather information on sociodemographic and demographic data, including age, socioeconomic status, education, occupation, religion, caste/tribe, lifestyle indicators and migration status. Data on socioeconomic position (SEP) was collected through a subset of questions used in the Standard of Living Index, which is household-level, asset-based scale devised for India).[19,20] SEP was calculated for both current status and childhood status by summarizing the weighted response scores as recommended for the Standard of Living Index.[19] The full Standard of Living Index has a large number of items (29 in total), but we used 14 items (quality of house; toilet facilities; source of lighting, drinking water; land ownership; possession of clock, radio, television, bicycle, motorcycle, car, tractor, refrigerator, telephone), keeping the ones we believed to be most informative for our study population. Measurement at the household level is appropriate in the Indian context, in which the individual's SEP has less impact on their material wealth. This asset-based score was considered a more appropriate indicator of SEP for these analyses than education, income, or occupation alone, because it is more likely to reflect the changes that migrants experience following their move to urban areas. In the context of developing countries, low SLI is associated with tobacco use[21] and with mortality (Subramanian *et al*. 2006b), indicating its validity as a socioeconomic marker. For each residence, participants were asked to report if the place was a village, town, small city or large city, guided where necessary by criteria defined by the Indian Census.[22] Other covariates considered for this study were background characteristics such as age, education, current marital status, religion, caste/tribe status, occupation, and SEP, self-perceived current health status, and preferred choice of living [Table 1].

### Statistical analysis

All statistical analyses were conducted using STATA software version 10 (StataCorp 2009; Stata Statistical Software: Release 10. College Station, TX: StataCorp LP). Standard descriptive analysis was done using Pearson's Chi-square test. We first examined sociodemographic differentials and premigratory and migration related experiences in the prevalence of psychological distress among the migrants just after migration and after settlement. Associations between psychological distress and various covariates were analyzed using multivariate logistic regression models. The analysis is based on 2112 rural to urban migrants aged ≥18 years which has been extracted from the total IMS sample of 7067 who reported their reasons for migration. The analysis was done separately for men and women as it was found that there is a strong evidence of gender differential in mental distress between men and women in our study both after migration and after settlement [Table 2].

### Ethics

Information sheets were translated into local languages and signed (or a witnessed thumbprint obtained if the participant was illiterate), and through this, informed consent was obtained. Ethics committee approval (including this process for obtaining informed consent) was obtained from the All India Institute of Medical Sciences Ethics Committee, reference number A-60/4/8/2004 and the London School of Hygiene and Tropical Medicine. The procedures followed were in accordance with the ethical standards of the committee.

## Results

### Profile of the migrants

Table 1 gives the sample distribution by selected characteristics of the migrants. The mean ages of men and women were 44.7 years (standard deviation [SD] ±8.6) and 39.5 years (SD ± 8.8), respectively. More than half (55%) had a senior secondary education and one out of five had graduate or professional degrees. Almost all were married and were Hindus and two out of four belong to the other category of caste/tribe. 90% of the migrant women were engaged in household works while more than half of the men were employed in skilled manual jobs. Current wealth status and childhood wealth status were almost similar with the exception that one out of five migrants belonged to the lowest category of SEP in their childhood. Better availability of services was the dominant reason for migration followed by better economic prospects and social reasons among the migrants. Furthermore 5% reported of other push factors. More than half of the migrants (55%) had already spent 25–50% of their lifetime in an urban area while half of them were living between 16 and 20 years in an urban area (mean ± SD: 20.0 ± 5.4). Half of the migrants were single at the time of migration and in 22% cases spouse joined migrants after 1 year. Two out of five migrants were accepted at their workplace after a few months of their migration while one out of three adjusted with the new urban life after a few months. More than half of the migrants reported that given a choice, they would have preferred to live in large cities while two out of five rated their current health status as good.

### Prevalence of psychological distress just after migration and after resettlement

Prevalence of mental distress just after migration was higher (7.3% [95% confidence interval (CI): 6.2–8.4]) than the prevalence after resettlement (4.7% [95% CI: 3.8–5.6]). The reasons for migration was associated with higher prevalence of psychological distress among the migrants both just after migration (*P* < 0.0001) and after settlement (*P* = 0.016). Prevalence of psychological distress was more than 3 times higher (14.8%) among those who reported push factor as a reason of migration, followed by pull factors such as social reasons (10.1%). Strong association between age and psychological distress was observed just after migration (*P* < 0.0001) but not after settlement (*P* = 0.247). Prevalence of psychological distress was almost 3 times higher (14.1%) in the age below 30 years than in age above 50 years. Psychological distress was more than 2 times higher (*P* < 0.0001) among women than among men both during just after migration and after settlement. Prevalence of psychological distress varied according to current occupation both just after migration (*P* < 0.001) and currently (*P* = 0.002). Psychological distress was almost double among the household workers (10.5%) than those who engaged in professional and semi-professional jobs. Current wealth status (household living standard) was also associated with higher psychological distress among the migrants just after migration but not after settlement. Migrants belonging to lowest wealth status household showed higher prevalence of psychological distress than migrant belonging to higher wealth status households. Non acceptance in workplace even after 1 year and not being able to adjust in the new urban environment after more than a year, show strong association with psychological distress both just after migration and after settlement. Prevalence of psychological distress was almost 6 times higher among those migrants who reported of not being accepted in their workplace even after more than a year than those who reported of being accepted immediately. Psychological distress was more than 6 times higher among migrants who reported of not being able to adjust in the new urban environment even after more than a year of their migration and resettlement. Psychological distress was more common among those who perceived their current health status as poor or very poor currently (7.5%) than who rated their current health status as very good.

### Associations between socioeconomic factors, migration experiences and psychological distress just after migration

After adjusting for all the potential confounders, the odds of prevalence of psychological distress was 6 times higher among men (odd ratio [OR]: 5.8; 95% CI: 1.89–17.68; *P* = 0.002) and women (OR: 6.3; 95% CI: 2.07–19.32; *P* = 0.001) who reported push factor as a reason for migration than those who reported pull factors such as better availability of services in urban areas as a reason [Table 3]. The odds of suffering from psychological distress was 16 times higher among men (OR: 16.4; 95% CI: 1.34–201.8; *P* = 0.029) and 6 times more among women (OR: 6.4; 95% CI: 2.12–19.29; *P* = 0.001) who reported that they still could not adjust in the new urban environment than those who immediately adjusted to the new environment. The odds of prevalence of psychological distress was higher among men if the spouse joined the migrant after more than a year (OR: 2.38) with reference to single migrants; for women if she reports of joining her husband within 6 months of migration (OR: 1.9). The association between other covariates and psychological distress just after migration was not found substantial among both men and women.

### Associations between socioeconomic factors, migration experiences and psychological distress after resettlement

Migrant men who reported push factor as a reason for migration were 4 times (OR: 4.3; 95% CI: 1.40–13.5; *P* = 0.011) more likely to suffer from psychological distress than who reported pull factors as a reason for migration [Table 3]. This association was not found among women. The odds of suffering from psychological distress was 5 times higher among men (OR: 5.1; 95% CI: 1.12–23.2; *P* = 0.035) and 6 times higher among women (OR: 5.6; 95% CI: 1.61–19.58; *P* = 0.007) who perceived their current health status as good with reference to those who perceived their current health status as very good. The association between other covariates and psychological distress after settlement was not found substantial among both men and women.

## Discussion

In the current investigation, we examined the association between sociodemographic characteristics, premigratory and migratory factors and psychological distress in migrants just after migration and long after their resettlement by exploring the data from the IMS. The study shows high prevalence of psychological distress in the migrant population just after migration and substantiate that push factor as a reason for migration and not being able to adjust in the new urban environment increased the risk of psychological distress among the rural to urban migrants in India. This relationship was strong and significantly higher among migrants during the time when they just migrated than today when they have resettled. Indeed, this is the first known cross sectional, population-based study to demonstrate this association in Indian rural to urban migrants and thus add to the limited data on the premigratory and migratory factors on the risk of developing psychological distress in developing countries. This finding integrates prior research demonstrating the acculturation stress hypothesis that stresses of living in a new culture promote mental disorder.[23]

Findings on prevalence of psychological distress such as depression across different ethnic and migrant populations are equivocal across the globe.[2] Studies in the west showed that migration and preemigration experiences have profound effects on mental health and that acculturation differences have deleterious effects on mental health and family functioning.[24] Studies based on clinical research and community studies have found that migrants who suffered emotional traumas are more likely to demonstrate psychological disorders.[25–28] It has been observed that migrants who were subjected to changed psychosocial environment in terms of low social support, changed patterns of social participation or lack of control over their life events in a new society, exhibit higher level of psychological symptoms.[29,30] Hence, it can be assumed that migration by itself does not constitute a threat to the health of migrants, but changes in psychosocial factors might be the important mediators in the pathway between migration and mental health status.[31–33] This might be the reason that studies dealing with acculturation have reported higher distress and depressive symptoms for those migrants who migrate to culturally and socially distinct societies and try to adapt to the new social circumstances after migration.[33–35] There are no or limited studies in developing countries on the course and outcome of psychological distress among the migrants but some studies found the prevalence of depression and anxiety among vulnerable population groups is much higher; for example, amongst persons displaced by the armed conflict in Nepal, the prevalence was found to be as high as 80%.[36] In India, overall, the point prevalence of serious mental disorders is about 10–20/1000 population.[37] Despite India's National Mental Health Programme which was introduced almost 30 years ago, provision of services are severely lacking. 20% of districts have implemented the District Mental Health Programme plan and only 10% of those who need urgent mental healthcare are receiving the required help with the existing services.[37,38] Moreover, huge disparity in access to mental health care exits as the concentration of facilities and services is greater in urban areas[37] and no facilities for migrant population exist as such.

Status-based discrimination and inequity have been associated with the process of migration, especially with economics-driven internal migration and our study shows that migrants stating push factors (such as social discrimination, absolute lack of livelihood opportunity in rural area, security reasons [personal/political], natural disaster [floods/drought], no clear reason/don't know, or any other reason) as a reason for migration were more vulnerable to the risk of mental distress than others. This finding integrates prior research where it was found that perceived social stigma and discriminatory experiences had direct negative effects on psychological distress and quality of life among rural-to-urban Chinese migrants.[39]

### Strength and limitations of the study

The strength of our study includes the large geographically representative data and use of sibling pair design which provides a high level of control for potential confounding factors and early life exposures. A major limitation of the study is that there is a risk of poor recall of the experiences just after migration, since half of our sample population had migrated 16–20 years before. It is thus difficult to ensure how accurate the respondents reported about how they felt immediately after migration 20 years ago. This might partly explain the low prevalence of psychological distress in the migrants in this study. Also, the prevalence rate for psychological distress in this study are more likely to be symptomatic rather than the actual rate since a clinical diagnosis to establish a true prevalence was not available. The questions assessing the psychological distress symptoms of the migrants were collected by self-reporting and thus raised the concerns about its validity. Our response rates were moderate which may have resulted in selection bias among those taking part in the study, but this would be unlikely to affect the associations observed between the exposure and outcome variable. However, self-reported health and related psychosocial variables are widely used in European[40–42] and American studies.[43,44]

From a methodological point of view, the weakness of the study is that it is based on a cross-sectional design. The inherent problem of a cross-sectional design is that the outcome (in this case psychological distress) and the exposure (in this case socioeconomic characteristics and premigratory and migratory experiences) are collected simultaneously and thereby preventing conclusions regarding causality. Also, we do not have data on the psychological health of the rural migrants in our sample prior to their migration to the urban area. Future studies in India should evaluate the development of psychological distress symptoms by sampling populations in migrants' place of origin. Moreover, less attention has been paid to the information bias emerging from the dependent error in the cross-sectional studies, which means a possible correlation between the degree of error in measured exposure and measured outcome. Thus, it is possible that estimated associations between sociodemographic characteristics and migration experiences and psychological distress are falsely inflated in our study.

## Conclusion

Internal migration is a major phenomenon in India and an important factor in the assessment of mental health planning and treatment in developing countries. Stressful experiences during migration appear to have long lasting effects on the mental health of rural to urban migrants which are evident in this study. This study provides some of the empirical evidence of an association between sociodemographic characteristics, migration experiences, and high psychological distress among the Indian migrants just after migration and after their resettlement in a developing country setting. Our findings suggest that causative and associative factors of psychological disorders/mental distress such as depression should be assessed in the context of the migration itself. There is a need to develop mental health intervention programs to deal with chronic mental distress to help the migrants live a healthy life. Moreover, an enhancement of quality of life and reduction of acculturation stress might be an effective intervening factor for preventive measures. Premigration training with a focus on the establishment of effective coping skills and preparation of migration may be helpful to improve their quality of life and mental health.

Migration remains an enigma for the clinician because not all migrants go through the same experiences and or settle in similar social circumstances. The process of migration and subsequent cultural and social adjustment and also an adjustment in their workplace thus play a key role in the mental health of the individual, which is evident in our study. Clinicians must take a range of these factors into account when assessing and planning intervention strategies aimed at the migrant individual and his or her social context. Further, to help promote the mental well-being of migrants, policy makers and community health providers can work to ensure that mental health coverage is available at primary health care centers and community/private health clinics where migrants receive their care. In addition, health care providers can also be encouraged to ask new migrants how stressful their move to the urban area has been and how they are adjusting, and should routinely screen for anxiety and depression symptoms using short, effective diagnostic tools. Finally, community health care providers and other organizations can take steps to help the new migrants develop strategies to adjust with the new urban environment and find strength in their cultural heritage, families, and broader social networks.

## Figures and Tables

**Table 1:** Sample distribution (%) by selected characteristics of the migrants (*n*=2112) in the IMS, 2005-2007

Characteristics of migrants	Men (%)	Women (%)	Total (%)	*n*
Age of migrants				
<30	3.4	17.1	9.8	206
30-39	23.9	25.4	24.6	519
40-49	36.7	44.7	40.5	855
>50	35.9	12.9	25.2	532
Mean±SD)	44.7±8.6	39.49±8.8	42.3±9.1	
Education*				
No education	1.1	20.1	9.9	210
Primary	3.7	24.5	13.4	283
Senior secondary	65.0	42.7	54.6	1154
Graduate and professional	30.2	12.7	22.0	465
Current marital status				
Single	1.1	0.0	0.6	12
Married	98.3	97.9	98.1	2072
Widow/widower	0.6	2.1	1.3	28
Religion				
Hindu	94.6	91.9	93.3	1971
Non-Hindu	5.4	8.1	6.7	141
Caste/tribe status†				
Scheduled caste	16.7	20.9	18.7	394
Scheduled tribes	5.3	5.4	5.4	113
Other backward caste	35.9	32.1	34.1	720
Other	42.1	41.6	41.6	884
Occupation				
Household work	0.4	89.6	42.3	901
Unemployed/unskilled/semiskilled manual	4.3	2.1	3.3	70
Skilled manual	56.8	2.8	31.5	666
Professional/semiprofessional	38.6	5.4	23.1	488
Current standard of living‡				
Lowest	33.3	33.1	33.2	701
Middle	31.1	34.6	32.3	692
Highest	35.6	32.3	34.0	719
Childhood standard of living‡				
Lowest	47.6	37.1	42.7	902
Middle	37.0	35.0	36.1	762
Highest	15.4	27.9	21.2	448
Reason for migration				
Pull factors such as				
Better availability of services	45.7	21.9	34.6	731
Better economic prospects/promotion in urban area	45.5	12.2	30.0	633
Social reasons (to be with family/friends/marriage migration)	2.0	62.0	30.0	633
Push factors§	6.8	3.9	5.5	115
Percentage of life lived in an urban area				
0-25	6.3	3.4	4.9	104
25-50	53.3	56.7	54.9	1159
50-75	36.1	27.9	32.3	682
75-100	4.3	12.1	7.9	167
Spouse joined migrant				
Single at the time of migration	50.2	51.0	50.6	1068
Within 6 months	18.6	17.8	18.2	385
Between 7-12 months	8.3	8.7	8.5	179
After 1-year	22.9	22.5	22.7	480
Acceptance in workplace				
Immediately	16.7	5.0	11.2	237
After few weeks	25.3	8.9	17.7	373
After few months	39.5	10.0	25.7	542
After more than a year/still do not accept	16.7	4.5	11.0	232
Not applicable/not working	1.8	71.7	34.4	726
Adjustment in the urban environment				
Immediately	18.3	12.8	15.7	332
After few weeks	27.4	19.4	23.7	499
After few months	36.2	42.1	39.0	822
After more than a year/still do not accept	18.1	25.7	21.7	457
Current choice of living				
Village	45.9	28.7	37.9	800
Town	5.8	5.8	5.8	123
Small city	3.3	4.0	3.6	76
Large city	45.1	61.4	52.7	1113
Self-perception of current health				
Very good	22.1	15.5	19.0	402
Good	43.7	41.3	42.6	899
Average	29.6	31.7	30.8	650
Poor/very poor	4.6	11.1	7.6	161
Total	1127	985	100.0	2112

* Education: No education (0 years of education), primary (1-5 years of education), senior secondary (6-10 years of education), graduate and professionals (10+ years of education)

† Scheduled castes and scheduled tribes are identified by the Government of India as socially and economically backward and needing protection from social injustice and exploitation. Other backward class is a diverse collection of intermediate castes that were considered low in the traditional caste hierarchy but are clearly above scheduled castes. Others are thus a default residual group that enjoys higher status in the caste hierarchy

‡ The current and childhood SLI was calculated by applying standard weights to subsets of questions from a household level asset-based scale devised for Indian surveys, and rescaling them to the full score. The items were: Quality of house; toilet facilities; source of lighting, drinking water; land ownership; possession of clock, radio, television, bicycle, motorcycle, car, tractor, refrigerator, telephone. The score was then categorised into tertiles to produce low, medium and high SEP groups

§ Push factors for migration in this study were absolute lack of livelihood opportunity in rural area, social discrimination, personal security (personal/political reasons), natural disaster (floods/drought), no clear reason/don't know, any other reason. IMS: Indian Migration Study, SLI: Standard of living, SEP: Socioeconomic position, SD: Standard deviation

**Table 2:** Percentage prevalence of psychological distress just after migration and after resettlement, currently among the migrants in the IMS 2005-2007

Characteristics of migrants	Just after migration		After resettlement	
	Psychological distress	*χ*^2^*P*	Psychological distress	*χ*^2^
Age of migrants				
<30	14.1	<0.0001	3.4	0.247
30-39	8.5		4.1	
40-49	6.1		5.9	
>50	5.5		4.1	
Sex of migrant				
Male	4.5	<0.0001	3.2	<0.0001
Female	10.5		6.5	
Education				
No education	9.5	0.067	5.7	0.023
Primary	8.8		8.1	
Senior secondary	7.5		4.1	
Graduate and professional	4.7		3.9	
Current marital status				
Single	8.3	0.741	8.3	0.699
Married	7.3		4.7	
Widow/widower	3.6		7.1	
Religion				
Hindu	7.5	0.271	4.9	0.272
NonHindu	5.0		2.8	
Caste/tribe status				
Scheduled caste	5.1	0.270	4.6	0.617
Scheduled tribes	8.9		4.4	
Other backward caste	8.1		4.0	
Others	7.5		5.4	
Occupation				
Household work	10.5	<0.0001	6.8	0.002
Unemployed/unskilled/semiskilled manual	5.7		1.4	
Skilled manual	4.7		3.0	
Professional/semi-professional	5.3		3.9	
Current wealth status				
Lowest	9.3	0.003	4.0	0.345
Middle	8.0		5.6	
Highest	4.7		4.6	
Childhood wealth status				
Lowest	6.0	0.134	4.0	0.126
Middle	8.4		4.6	
Highest	8.0		6.5	
Reason for migration				
Better availability of services	4.7	<0.0001	3.3	0.016
Better economic prospects/promotion in urban area	6.2		4.1	
Social reasons (to be with family/friends/marriage migration)	10.1		6.6	
Push factors	14.8		7.0	
Percentage of life lived in an urban area				
0-25	6.7	0.053	4.8	0.901
25-50	6.0		4.8	
50-75	9.1		5.0	
75-100	9.6		3.6	
Spouse joined migrant				
Single at the time of migration	7.2	0.297	5.7	0.158
Within 6 months	9.4		4.4	
Between 7-12 months	5.6		3.4	
After 1-year	6.5		3.3	
Acceptance in workplace				
Immediately	1.7	<0.0001	3.0	0.051
After few weeks	5.1		3.2	
After few months	6.8		3.9	
After more than a year/still do not accept	7.8		6.0	
Not applicable/not working	10.2		6.3	
Adjusted in the urban environment				
Immediately	2.1	<0.0001	4.8	0.008
After few weeks	3.8		4.0	
After few months	7.7		3.5	
After more than a year/still do not accept	13.8		7.7	
Choice of living				
Village	7.5	0.001	4.6	0.804
Town	15.5		6.5	
Small city	10.5		4.0	
Large city	6.0		4.7	
Self-perception of current health				
Very good	7.2	0.021	1.2	<0.0001
Good	9.0		6.8	
Average	6.0		3.4	
Poor/very poor	3.1		7.5	
Total percentage	7.3		4.7	
Total number	154		100	

IMS: Indian Migration Study

**Table 3:** Adjusted association (ORs and 95% CI) of socioeconomic and demographic characteristics and migration experiences on psychological distress among men and women just after migration and after settlement (*n*=2112), IMS 2005-2007

Characteristics of migrants	OR (95% CI)			
	Psychological distress just after migration		Psychological distress after resettlement	
	Men	Women	Men	Women
Age of migrants				
<30^R^	1	1	1	1
0-39	0.56 (0.15-2.13)	0.76 (0.33-1.74)	0.51 (0.10-3.22)	1.44 (0.34-6.04)
40-49	0.50 (0.11-2.26)	0.55 (0.21-1.42)	0.66 (0.10-4.47)	2.59 (0.59-11.29)
>50	0.67 (0.13-3.30)	0.80 (0.24-2.61)	0.63 (0.10-4.80)	2.33 (0.43-12.67)
Education				
No education^R^	1	1	1	1
Primary	0.94 (0.61-10.55)	1.13 (0.54-2.36)	0.10 (0.00-10.23)	1.44 (0.62-3.32)
Senior secondary	0.65 (0.13-5.56)	0.99 (0.49-2.00)	0.15 (0.14-15.20)	0.83 (0.34-2.06)
Graduate and professional	0.49 (0.99-8.47)	0.33 (0.11-1.02)	0.24 (0.22-7.89)	0.16 (0.03 (0.97)
Current marital status				
Single^R^	1	1	1	1
Married	0.89 (0.10-9.34)	8.66 (0.79-9.49)	0.54 (0.04-7.40)	18.35 (0.91-37.20)
Widow/widower	-	-	-	-
Religion				
Hindu^R^	1	1	1	1
NonHindu	-	0.67 (0.25-1.75)	1.40 (0.29-6.88)	0.24 (0.05-1.05)
Caste/tribe status				
Scheduled caste^R^	1	1	1	1
Scheduled tribes	1.88 (0.24-14.50)	2.59 (0.88-7.59)	1.57 (0.23-10.72)	0.62 (0.15-2.60)
Other backward caste	2.36 (0.68-8.20)	1.59 (0.78-3.26)	1.57 (0.23-4.03)	0.88 (0.40-1.97)
Others	2.57 (0.76-8.75)	1.07 (0.53-2.16)	1.18 (0.35-4.00)	0.79 (0.37-1.67)
Occupation				
Household work^R^	1	1	1	1
Unemployed/unskilled/semi-skilled manual	-	5.74 (1.23-26.87)	-	-
Skilled manual	0.38 (0.04-3.64)	0.91 (0.20-4.18)	0.18 (0.01-3.40)	0.04 (0.00-1.42)
Professional/semiprofessional	0.35 (0.04-3.36)	1.09 (0.29-4.08)	0.11 (0.01-2.20)	1.70 (0.34-8.53)
Current wealth status				
Lowest^R^	1	1	1	1
Middle	1.67 (0.67-4.20)	1.11 (0.61-2.02)	1.10 (0.38-3.18)	1.09 (0.52-2.31)
Highest	1.76 (0.65-4.76)	0.44 (0.20-0.97)	1.68 (0.57-4.97)	0.79 (0.33-1.89)
Childhood wealth status				
Lowest^R^	1	1	1	1
Middle	1.03 (0.49-2.17)	1.44 (0.80-2.56)	2.11 (0.88-5.09)	0.88 (0.43-1.81)
Highest	1.18 (0.44-3.15)	1.17 (0.59-2.34)	2.47 (0.85-7.18)	1.74 (0.80-3.80)
Reason for migration				
Better availability of services^R^	1	1	1	1
Better economic prospects/promotion in urban area	1.66 (0.79-3.50)	1.43 (0.58-3.51)	1.83 (0.80-4.18)	1.50 (0.54-4.15)
Social reasons (to be with family/friends/marriage migration)	1.15 (0.14-9.57)	1.73 (0.89-3.35)	1.27 (0.08-19.52)	2.15 (0.99-4.67)
Push factors	5.77 (1.89-17.68)	6.32 (2.07-19.32)	4.33 (1.40-13.45)	0.51 (0.06-4.57)
Percentage of life lived in an urban area				
0-25^R^	1	1	1	1
25-50	0.74 (0.19-2.83)	1.38 (0.28-6.71)	0.98 (0.25-3.88)	1.15 (0.22-5.90)
50-75	1.86 (0.46-7.74)	1.37 (0.27-7.04)	0.79 (0.17-3.58)	2.10 (0.38-11.56)
75-100	1.37 (0.18-10.36)	1.21 (0.20-7.41)	1.27 (0.19-8.45)	1.19 (0.12-12.22)
Spouse joined migrant				
Single at the time of migration^R^	1	1	1	1
Within 6 months	2.75 (1.21-6.23)	1.91 (1.03-3.53)	0.59 (0.20-1.75)	0.77 (0.36-1.66)
Between 7-12 months	0.31 (0.04-2.47)	1.69 (0.70-4.05)	0.22 (0.03 (1.81)	0.41 (0.14-1.20)
After 1-year	2.38 (0.94-6.05)	1.26 (0.62-2.60)	0.82 (0.29-2.27)	0.21 (0.08-0.55)
Acceptance in workplace				
Immediately^R^	1	1	1	1
After few weeks	0.78 (0.12-4.93)	6.28 (0.84-46.99)	1.53 (0.31-7.63)	0.93 (0.15-5.82)
After few months	0.77 (0.13-4.68)	4.41 (0.64-30.21)	1.62 (0.35-7.58)	2.10 (0.42-10.56)
After more than a year/still do not accept	1.27 (0.18-8.81)	2.96 (0.37-23.83)	4.88 (0.93-25.53)	1.49 (0.24-9.11)
Not applicable/not working	10.93 (1.29-9.25)	2.10 (0.33-13.54)	2.43 (0.12-48.59)	1.05 (0.25-4.46)
Adjustment in the urban environment				
Immediately^R^	1	1	1	1
After few weeks	8.54 (0.75-9.78)	0.38 (0.10-1.49)	1.18 (0.29-4.91)	0.51 (0.17-1.50)
After few months	15.09 (1.33-17.7)	2.11 (0.70-6.33)	0.83 (0.19-3.55)	0.47 (0.18-1.21)
After more than a year/still do not accept	16.42 (1.33-20.2)	6.40 (2.12-19.29)	0.40 (0.10-2.10)	1.38 (0.57-3.38)
Choice of living				
Village^R^	1	1	1	1
Town	0.69 (0.18-2.64)	3.01 (1.31-6.95)	1.67 (0.45-6.06)	1.34 (0.38-4.79)
Small city	2.05 (0.54-7.84)	0.68 (0.19-2.41)	0.82 (0.10-7.38)	0.47 (0.08-2.77)
Large city	0.45 (0.22-0.94)	1.01 (0.60-1.74)	0.68 (0.30-1.53)	1.28 (0.67-2.47)
Self-perception of current health				
Very good^R^	1	1	1	1
Good	0.49 (0.20-1.15)	2.12 (1.05-4.27)	5.09 (1.12-23.22)	5.62 (1.61-19.57)
Average	0.75 (0.28-1.96)	2.12 (1.05-4.26)	2.64 (0.48-14.48)	3.40 (0.89-13.00)
Poor/very poor	-	0.38 (0.12-1.21)	4.40 (0.54-36.01)	5.70 (1.39-23.41)
Number of respondents	2109	2109	2109	2109

OR could not be analyzed due to small number of cases in the cell. R: Reference category, OR: Odd ratio, CI: Confidence interval, IMS: Indian Migration Study

## Appendix 1

The push factors are those that compel a person, due to different reasons, to leave that place and go to some other place, for instance, low productivity, unemployment and underdevelopment, poor economic conditions, lack of opportunities for advancement, exhaustion of natural resources and natural calamities may compel people to leave their native place in search of better economic opportunities. The non-availability of alternative sources of income (non-agricultural activities) in rural areas is also important factor for migration. In addition to this, the existence of the joint family system and laws of inheritance, which do not permit the division of property, may also force many young men to move out to cities in search of jobs. Even sub division of property leads to migration, as the property become too small to support a family.

The Pull factors refer to those factors which attract the migrants to an area, such as, opportunities for better employment, higher wages, facilities, better working conditions and amenities etc. There is generally city ward migration, when rapid growth of industry, commerce and business takes place. Migration from the country side to the cities bears a close functional relation to the process of industrialization, technological advancement and other cultural changes which characterize the evolution of modern society in almost all parts of the world. Under the capitalistic model of development, there is a tendency for large proportion of investments to concentrate in the urban centers which encourage people to move to urban areas in the expectation of higher paid jobs. Thus, pull factors operate not only in the rural-urban migration, but also in other types of domestic as well as international migration.

## References

[1] Schwarzweller HK, Seggar JF (1967). Kinship involvement: A factor in the adjustment of rural migrants. J Marriage Fam.

[2] Bhugra D, Jones P (2001). Migration and mental illness. Advances in Psychiatric Treatment.

[3] Carta MG, Bernal M, Hardoy MC, Haro-Abad JM, Report on the Mental Health in Europe Working Group (2005). Migration and mental health in Europe (the state of the mental health in Europe working group: Appendix 1). Clin Pract Epidemiol Ment Health.

[4] Odegaard O (1932). Emigration and insanity. Acta Psychiatrica et Neurologica Scandinavica.

[5] Pope HG, Ionescu-Pioggia M, Yurgelun-Todd D (1983). Migration and manic-depressive illness. Compr Psychiatry.

[6] Grove W, Clayton PJ, Endicott J, Hirschfeld RM, Andreasen NC, Klerman GL (1986). Immigration and major affective disorder. Acta Psychiatr Scand.

[7] Bhugra D (2004). Migration and mental health. Acta Psychiatr Scand.

[8] United Nations (2006). United Nations High Commission for Refugees. http://www.unhcr.org/cgi-bin/texis/vtx/home.

[9] Kimura SP, Mikolashek PL, Kirk SA (1975). Madness in paradise: Psychiatric crises among newcomers in Honolulu. Hawaii Med J.

[10] Hull D (1979). Migration, adaptation, and illness: A review. Sci Med Med Psychol Med Sociol.

[11] Eagles JM (1991). The relationship between schizophrenia and immigration. Are there alternatives to psychosocial hypotheses?. Br J Psychiatry.

[12] Papadopoulos I, Lees S, Lay M, Gebrehiwot A (2004). Ethiopian refugees in the UK: Migration, adaptation and settlement experiences and their relevance to health. Ethn Health.

[13] Syed HR, Dalgard OS, Dalen I, Claussen B, Hussain A, Selmer R (2006). Psychosocial factors and distress: A comparison between ethnic Norwegians and ethnic Pakistanis in Oslo, Norway. BMC Public Health.

[14] Lusome R, Bhagat RB (2006). Trends and Patterns of Internal Migration in India, 1971-2001.

[15] Lyngdoh T, Kinra S, Shlomo YB, Reddy S, Prabhakaran D, Smith GD (2006). Sib-recruitment for studying migration and its impact on obesity and diabetes. Emerg Themes Epidemiol.

[16] Ebrahim S, Kinra S, Bowen L, Andersen E, Ben-Shlomo Y, Lyngdoh T (2010). The effect of rural-to-urban migration on obesity and diabetes in India: A cross-sectional study. PLoS Med.

[17] Kinra S, Bowen LJ, Lyngdoh T, Prabhakaran D, Reddy KS, Ramakrishnan L (2010). Socio demographic patterning of non-communicable disease risk factors in rural India: A cross sectional study. Br Med J.

[18] Reddy KS, Prabhakaran D, Chaturvedi V, Jeemon P, Thankappan KR, Ramakrishnan L (2006). Methods for establishing a surveillance system for cardiovascular diseases in Indian industrial populations. Bull World Health Organ.

[19] International Institute for Population Sciences (IIPS) and ORC Macro (2000). National Family Health Survey (NFHS-2), 1998-99.

[20] Subramanian SV, Davey Smith G, Subramanyam M (2006). Indigenous health and socioeconomic status in India. PLoS Med.

[21] Subramanian SV, Nandy S, Irving M, Gordon D, Lambert H, Davey Smith G (2006). The mortality divide in India: The differential contributions of gender, caste, and standard of living across the life course. Am J Public Health.

[22] Registrar General and Census Commissioner, India (2006). Census of India.

[23] Breslau J, Aguilar-Gaxiola S, Borges G, Castilla-Puentes RC, Kendler KS, Medina-Mora ME (2007). Mental disorders among English-speaking Mexican immigrants to the US compared to a national sample of Mexicans. Psychiatry Res.

[24] Dunaev E (2012). Acculturation, Psychological Distress, and Family Adjustment among Russian Immigrants in the United States.

[25] Krupinski J, Burrows G (1986). The price of freedom: Young Indochinese Refugees in Australia.

[26] Gerrand V (1993). Mental Health Policy and Services for Women. Deakin Series in Public Policy and Administration No 4.

[27] Chung RC, Kawara-Singer M (1993). The Age of Migration: International Population Movements in Modern World.

[28] Chou KL, Wong WK, Chow NW (2011). Interaction between pre- and post-migration factors on depressive symptoms in new migrants to Hong Kong from Mainland China. Community Ment Health J.

[29] Silveira ER, Ebrahim S (1998). Social determinants of psychiatric morbidity and well-being in immigrant elders and whites in east London. Int J Geriatr Psychiatry.

[30] Mallett R, Leff J, Bhugra D, Pang D, Zhao JH (2002). Social environment, ethnicity and schizophrenia. A case-control study. Soc Psychiatry Psychiatr Epidemiol.

[31] Cochrane R, Bal SS (1987). Migration and schizophrenia: An examination of five hypotheses. Soc Psychiatry.

[32] Sashidharan SP (1993). Afro-Caribbeans and schizophrenia: The ethnic vulnerability hypothesis re-examined. Int J Psychiatry.

[33] Bhugra D (2004). Migration, distress and cultural identity. Br Med Bull.

[34] Black SA, Markides KS, Miller TQ (1998). Correlates of depressive symptomatology among older community-dwelling Mexican Americans: The Hispanic EPESE. J Gerontol B Psychol Sci Soc Sci.

[35] Wiking E, Johansson SE, Sundquist J (2004). Ethnicity, acculturation, and self reported health. A population based study among immigrants from Poland, Turkey, and Iran in Sweden. J Epidemiol Community Health.

[36] Thapa SB, Hauff E (2005). Psychological distress among displaced persons during an armed conflict in Nepal. Soc Psychiatry Psychiatr Epidemiol.

[37] Srinivasa Murthy R, Agarwaal SP, Goel DS, Ichhpujani RL (2004). The National Mental Health Programme: Progress and problems. Mental Health – An Indian Perspective, 1946-2003.

[38] Ministry of Health and Family Welfare, Government of India. (MOHFW) Mental Health Programme. http://www.indiagovin/sectors/health_family/mental_healthphp.

[39] Wang B, Li X, Stanton B, Fang X (2010). The influence of social stigma and discriminatory experience on psychological distress and quality of life among rural-to-urban migrants in China. Soc Sci Med.

[40] Heistaro S, Vartiainen E, Puska P (1996). Trends in self-rated health in Finland 1972-1992. Prev Med.

[41] Krause NM, Jay GM (1994). What do global self-rated health items measure?. Med Care.

[42] Power C, Matthews S, Manor O (1998). Inequalities in self-rated health: Explanations from different stages of life. Lancet.

[43] Grant MD, Piotrowski ZH, Chappell R (1995). Self-reported health and survival in the Longitudinal Study of Aging, 1984-1986. J Clin Epidemiol.

[44] Kaplan GA, Camacho T (1983). Perceived health and mortality: A nine year follow-up of the human population laboratory cohort. Am J Epidemiol.

